# Supplementary material for: Genome-wide patterns of local adaptation in Western European Drosophila melanogaster natural populations
Source: Sci Rep. 2018 Nov 1;8:16143. doi: 10.1038/s41598-018-34267-0 (PMC6212444; doi:10.1038/s41598-018-34267-0)

Supplementary Information

Genome-wide patterns of local adaptation in Western European *Drosophila melanogaster* natural populations

Lidia Mateo, Gabriel E. Rech, and Josefa González

Figure S1. Median based correction of F_{ST} per gene.

A) Each dot represents the maximum F_{ST} per gene with respect to the number of SNPs assigned to that gene. Each gene is colored according to the percentile that occupies in the F_{ST} distribution. There is a positive correlation between the maximum F_{ST} per gene and the number of SNPs assigned to it (R=0.37; p-val=2.2e-16), which is an indicative of gene length bias. Horizontal lines separate genes into16 bins containing genes with similar number of SNPs and lack of correlation with their maximum F_{ST} value. B) The scatter plot shows the result of the median based corrected Z score of the F_{ST} per gene (Z_{ST}). The correlation between F_{ST} per gene and number of SNPs and has been eliminated (R=0.011; p-val=0.19). Each gene is colored according to the percentile that occupies in the Z_{ST} distribution. After the correction, candidate differentiated genes (yellow, orange and red) are evenly distributed across bins of genes with varying number of SNPs.

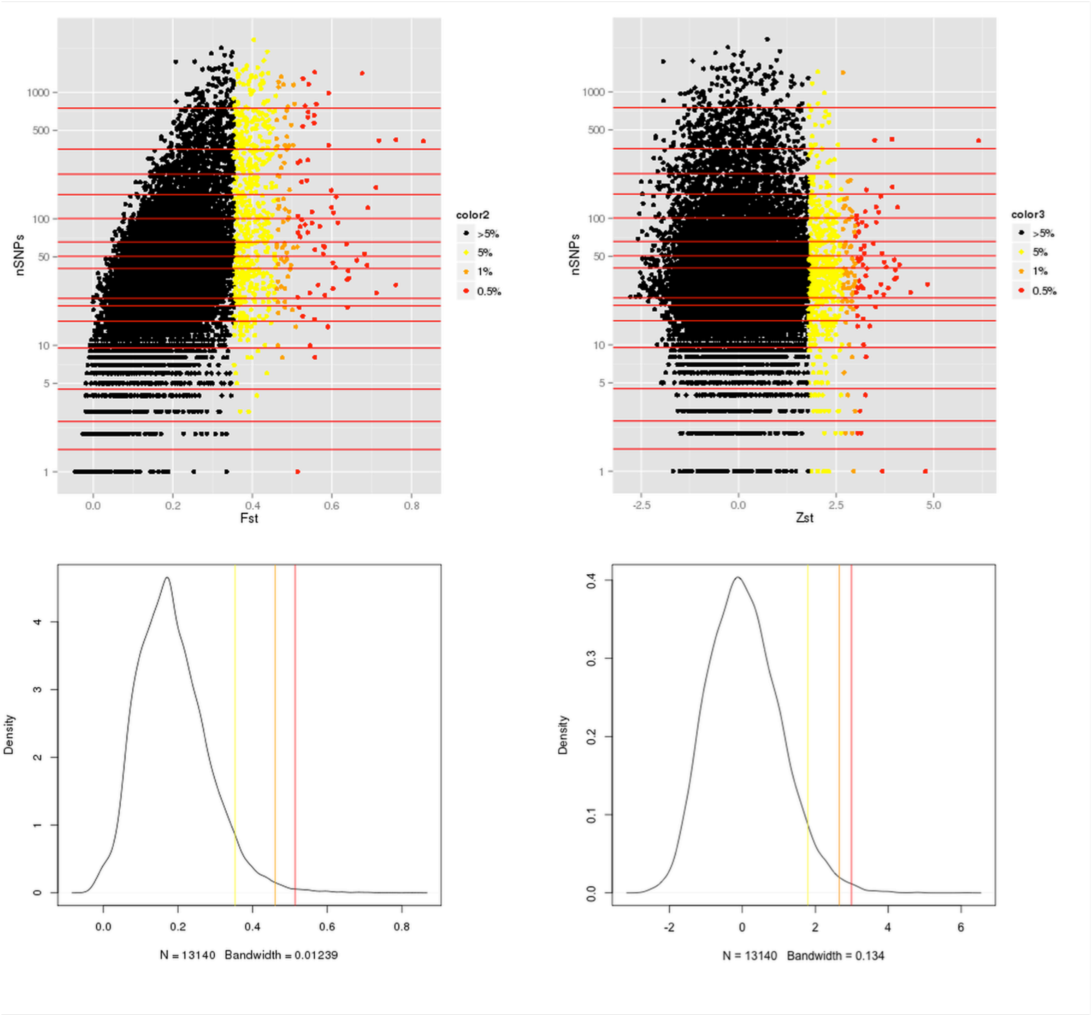

Figure S2. Genome-wide polymorphism (π) sampling one allele per genome for each chromosomal arm.

π estimates in 10.000 SNPs windows are plotted for each chromosome arm for each one of the four populations analyzed in this study.

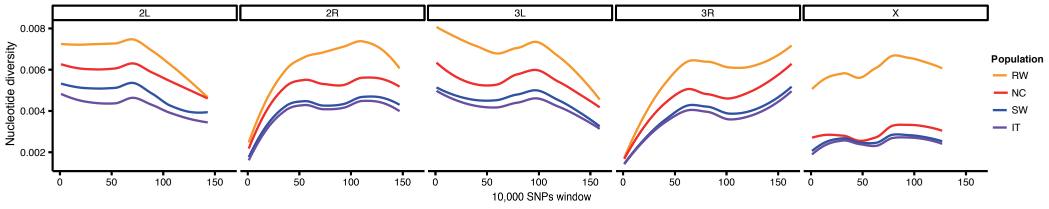

Figure S3. Maximum likelihood estimate of the number of ancestral populations according to *Admixture*.

The plot shows the cross validation errors estimated by *Admixture* with respect to the number of ancestral populations (k). Analysis with k = 2 and k = 3 gave the lowest cross-validation errors

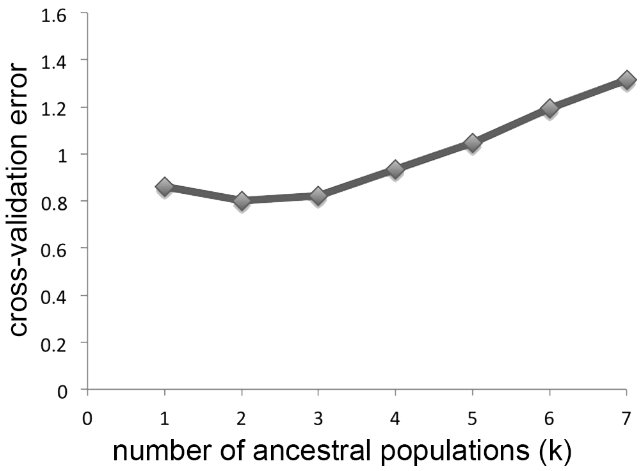

Figure S4. Genome-wide patterns of differentiation.

A) The empirical genome-wide F_{ST} distribution shows a maximum around zero followed by an exponential decay and a long tail of high F_{ST} values. Vertical lines indicate the top 5%, 1% and 0.5% percentiles of the distribution. B) The boxplot shows that the levels of differentiation are significantly heterogeneous among chromosomal arms (ANOVA p-value <2e-16). C) Visual representation of the genomic location of candidate differentiated SNPs falling in the top 5%, top 1% or top 0.05% tails of the empirical F_{ST} distribution of each chromosomal arm, which are shown in yellow, orange and red, respectively. Blue boxes indicate the chromosomal location of four common cosmopolitan inversions: In(2L)t , In(2R)NS, In(3L)P and In(3R)P.

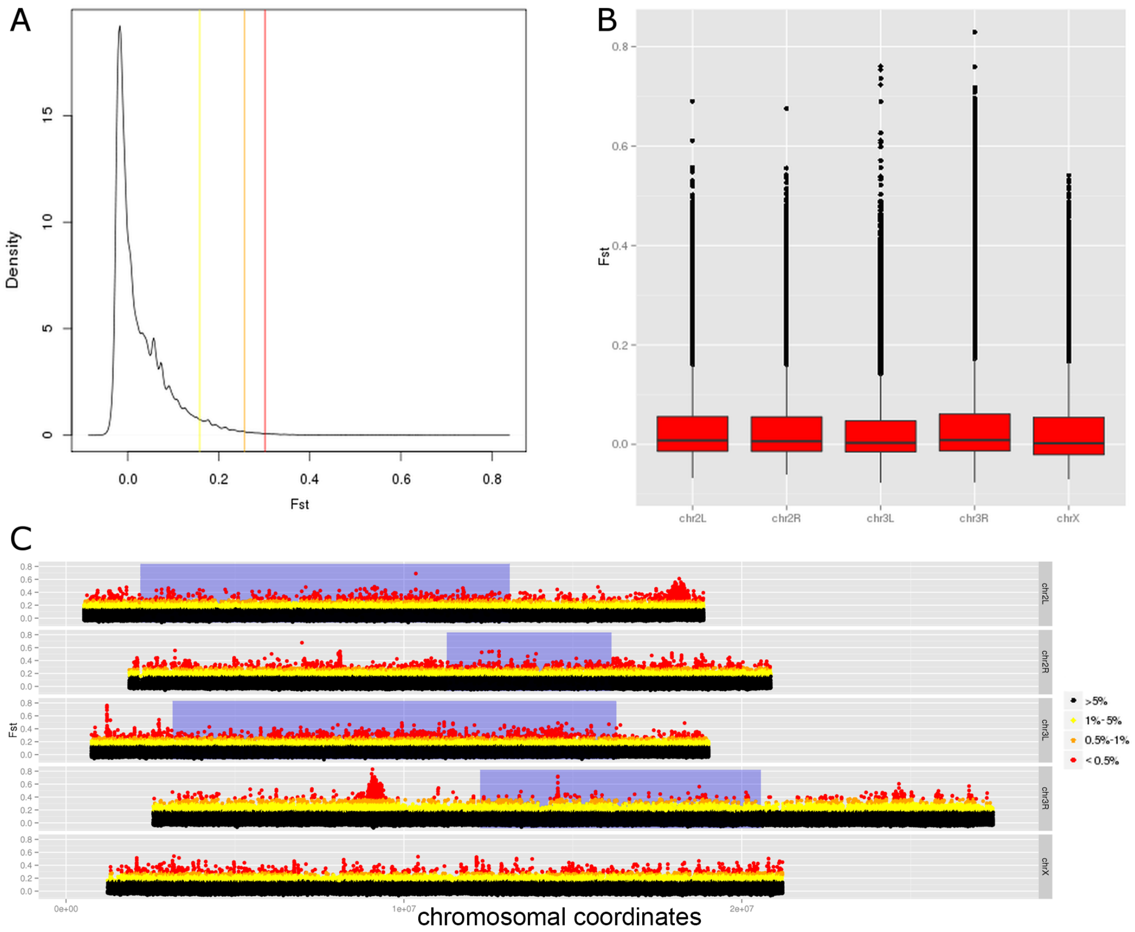

Figure S5. Empirical distributions of pairwise F_{ST} values for TEs and SNPs.

Kernel density peaks indicate where the F_{ST} values are concentrated.

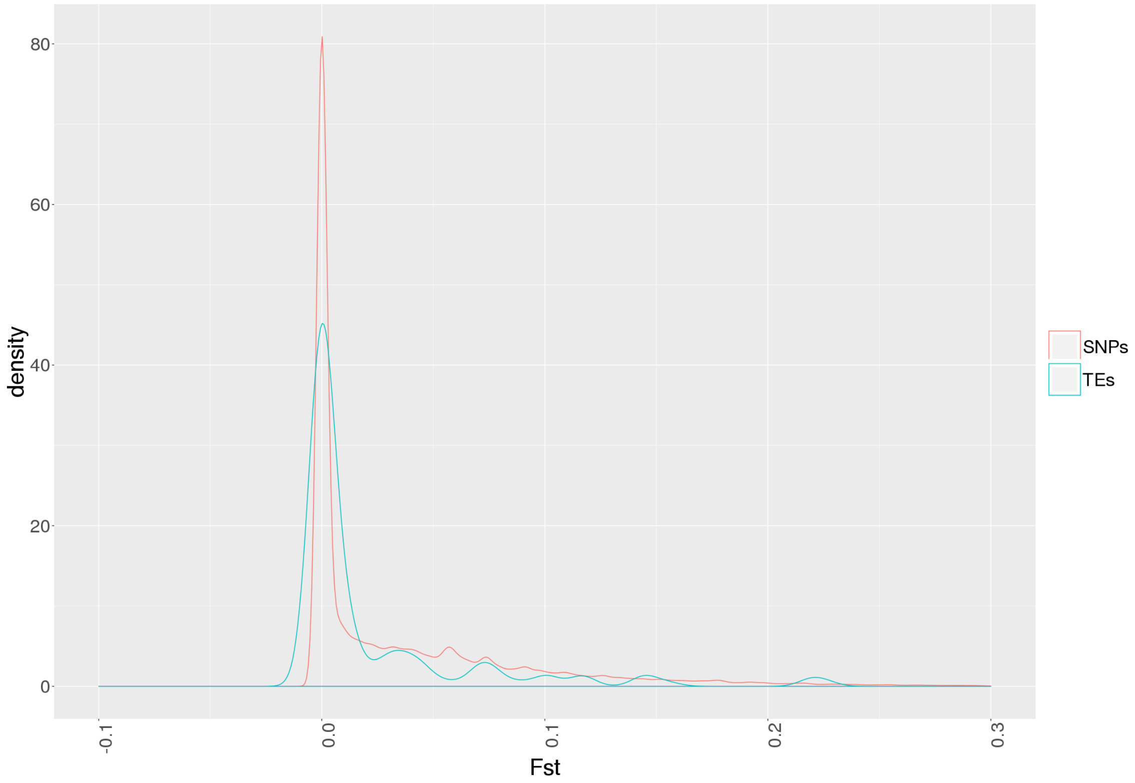

Supplement: Supplementary file 1 — Supplementary information [file 41598_2018_34267_MOESM1_ESM.pdf]
